# Validation of EpiTRAQ, a transition readiness assessment tool for adolescents and young adults with epilepsy

**DOI:** 10.1002/epi4.12427

**Published:** 2020-08-12

**Authors:** Sarah J. Clark, Nicholas J. Beimer, Acham Gebremariam, Linda L. Fletcher, Anup D. Patel, Lorrie Carbone, Jamie A. Guyot, Sucheta M. Joshi

**Affiliations:** ^1^ Susan B. Meister Child Health Evaluation and Research Center University of Michigan Ann Arbor MI USA; ^2^ Department of Neurology Michigan Medicine Ann Arbor MI USA; ^3^ Division of Pediatric Neurology Michigan Medicine Ann Arbor MI USA; ^4^ Division of Pediatric Neurology Nationwide Children’s Hospital Columbus OH USA

**Keywords:** epilepsy transition, neurology transition, pediatric to adult transition, validated transition readiness

## Abstract

**Objective:**

To design and validate a transition readiness assessment tool for adolescents and young adults with epilepsy and without intellectual disability.

**Methods:**

We adapted a general transition readiness assessment tool (TRAQ) to add epilepsy‐relevant items based on concepts in current epilepsy quality measures. The adapted tool, EpiTRAQ, maintained the original structure and scoring system. Concurrent with clinical implementation in pediatric and adult epilepsy clinics at an academic medical center, we assessed the validity and reliability of this adapted tool for patients 16‐26 years of age. This process included initial validation with 302 patients who completed EpiTRAQ between October 2017 and May 2018; repeat validation with 381 patients who completed EpiTRAQ between June 2018 and September 2019; and retest reliability among 153 patients with more than one completed EpiTRAQ.

**Results:**

Mean scores were comparable between initial and repeat validation populations (absolute value differences between 0.05 and 0.1); internal consistency ranged from good to high. For both the initial and repeat validation, mean scores and internal consistency demonstrated high comparability to the original TRAQ validation results. Upon retest, few patients rated themselves with a lower score, while the majority rated themselves with higher scores.

**Significance:**

EpiTRAQ is a valid and reliable tool for assessing transition readiness in adolescents and young adults with epilepsy and without intellectual disability.

**Key Point**

- A condition‐specific transition readiness assessment tool is important to support the unique needs of adolescents and young adults with epilepsy.
- EpiTRAQ was adapted from a well‐validated general assessment tool, with additional items reflecting key concepts in epilepsy quality measures.
- Concurrent with clinical implementation, we assessed the validity and reliability of EpiTRAQ with patients 16‐26 years of age.
- EpiTRAQ is a valid and reliable tool for assessing transition readiness in adolescents and young adults with epilepsy and without intellectual disability.

## 1 INTRODUCTION

Improvements in treatment and overall management of childhood epilepsy have led to more children and adolescents with epilepsy entering adulthood. Transition refers to the complex process of preparing patients and their families to move from the pediatric setting to the adult system of care. Although a structured program of transition has been recommended for children and youth with special health needs for nearly two decades,^1^ a recent consensus article underscores the paucity of evidence for successful transition of adolescents with epilepsy.^2^

A key strategy to support transition involves the use of structured transition readiness assessments to guide the process of helping youth gain experience and confidence in their ability to manage their own health care.^3^ Repeated administration of transition readiness assessments over the teen years helps adolescents and young adults—as well as their providers and parents—to recognize the knowledge and skills they already possess, and to understand where additional information, explanation, or practice is warranted.

General transition readiness assessments are useful in addressing basic elements of self‐management of health and health‐related skills. However, youth with epilepsy need additional condition‐specific knowledge and information that are not covered in general assessments.^4^, ^5^ At the same time, assessment tools must be brief and easy to implement in the clinical setting in order to gain widespread use. Therefore, our objective was to design and validate a condition‐specific transition readiness assessment tool for administration to adolescents and young adults with epilepsy and without intellectual disability in the clinical setting.

## 2 METHODS

### 2.1 Tool development

We had three key considerations in developing the epilepsy‐specific tool. First, to minimize the burden of implementation in the clinical setting, we wanted a tool that could be completed in a short amount of time (eg, in the waiting room prior to an appointment) with minimal staff assistance. Second, our plans included eventual adoption of the tool into our institution's electronic health record (EHR), so we wanted a tool that could easily be converted to that format. Third, we wanted to build on the experience of our institutional colleagues in administering transition readiness assessments.

In exploring these considerations, we found that colleagues had a positive experience using the Transition Readiness Assessment Questionnaire^6^ (TRAQ), a patient‐reported assessment tool designed for youth with special healthcare needs. The TRAQ includes 20 items, organized into five subscales: managing medications, appointment keeping, tracking health issues, talking with providers, and managing daily activities. Each TRAQ item is scored on a scale of 1‐5, representing the lowest level to highest level of readiness, from least to most independent based on the following fixed‐choice responses: 1 = No I don't know how; 2 = No but want to learn; 3 = No but learning; 4 = Yes have started; and 5 = Yes always do this when I need to.

The psychometric properties of TRAQ have been reported previously. ^7^, ^8^ An evaluation of ten transition assessment instruments named TRAQ as the best‐validated tool and the only tool with adequate content validity, construct validity, and internal consistency.^9^

We determined that TRAQ offered the option of modification for use in epilepsy clinics, while maintaining the core TRAQ structure for use in other clinical settings. This dual‐option structure would facilitate the future EHR adoption, as our epilepsy‐specific tool would utilize the same response format as a general tool used in other clinics. Therefore, we structured our new epilepsy‐specific tool as an expansion of TRAQ, maintaining all 20 questions, the fixed‐choice response options, and organization into five subscales.

To determine the epilepsy‐specific modifications, we reviewed the 2014 and 2017 American Academy of Neurology (AAN) epilepsy quality measurement set updates^10^, ^11^ led by one team member who was involved in the development of those measures (AP). We selected fifteen elements that were pertinent to adolescent care and important for proper transition and worked through several revisions to craft item wording that would both reflect the AAN measure descriptions and fit with the response options of TRAQ. We placed each new item into one of the TRAQ subscales. We referred to this modified, epilepsy‐specific instrument as EpiTRAQ.

### 2.2 Construct validity

The AAN quality measures aim to improve the delivery of care and outcomes for patients with epilepsy; as such, the new EpiTRAQ questions have strong construct validity with regard to the care and well‐being of youth with epilepsy. Table 1 presents the relationship between AAN quality measurement topics and the new epilepsy‐specific questions.

**TABLE 1:** Correspondence of EpiTRAQ questions with American academy of neurology quality measurement topics

AAN Quality Measure	New EpiTRAQ question (*Subscale Placement)*
Seizure frequency for patients with epilepsy^a^	Do you know how to keep track of your seizures? *Subscale: Tracking Health Issues*
Counseling for women of childbearing potential with epilepsy^a^	Can you describe how seizures can affect your ability to have children? [asked only for female patients] *Subscale: Tracking Health Issues* If you become pregnant, can you explain how seizures affect your pregnancy? [asked only for female patients] *Subscale: Managing Daily Activities* Can you explain how seizure medications and birth control medications affect one another? *Subscale: Managing Daily Activities*
Seizure intervention specified at each encounter^b^	Can you name your seizure medications? *Subscale: Managing Medications* Do you know what to do if you know that you are going to have a seizure? *Subscale: Tracking Health Issues* Do you know how to use rescue medications to stop a long or back‐to‐back seizure? *Subscale: Managing Medications* Do you know how a seizure action plan is used? *Subscale: Tracking Health Issues*
Etiology, seizure type, and epilepsy syndrome specified at each encounter^b^	Can you explain what type of seizures you have? *Subscale: Tracking Health Issues* Can you explain why you have epilepsy? *Subscale: Tracking Health Issues*
Querying and intervention for side effects of antiseizure therapy specified at each encounter^b^	Can you describe how epilepsy and seizure medications affect bone health? *Subscale: Managing Daily Activities* Can you describe how other medications and alcohol affect your seizure medications? *Subscale: Managing Daily Activities*
Personalized epilepsy safety issue and education provided yearly^b^	Can you explain how epilepsy affects school or having a job? *Subscale: Managing Daily Activities* Can you explain activities that you are not allowed to do? *Subscale: Managing Daily Activities* Can you describe how long you have to be seizure free before you are allowed to drive a car? *Subscale: Managing Daily Activities*

^a^ From AAN 2017 quality measurement topics.

^b^ From AAN 2014 quality measurement topics.

As an additional gauge of validity, the EpiTRAQ instrument was reviewed by two youth with epilepsy and a parent of an adolescent with epilepsy. All three felt that EpiTRAQ was easy to understand and complete; they recommended no changes.

### 2.3 Implementation of EpiTRAQ administration

EpiTRAQ was implemented in pediatric and adult neurology outpatient clinics at Michigan Medicine in Ann Arbor, Michigan. The Comprehensive Epilepsy Programs in the Departments of Neurology and Pediatrics at Michigan Medicine are Level 4 epilepsy programs, individually recognized by the National Association of Epilepsy Centers. The pediatric epilepsy program sees over 1700 children under 21 years of age for outpatient care each year, while the adult epilepsy program sees more than 3000 patients annually.

In October 2017, as part of a quality improvement project to improve transition support, pediatric and adult neurology clinics began requesting EpiTRAQ completion for established patients with a diagnosis of epilepsy who were attending scheduled appointments. The clinics targeted patients 16‐26 years of age, consistent with the age range for the original TRAQ. Implementation of EpiTRAQ and assessment of its reliability and validity were determined to be nonregulated quality improvement activity by the University of Michigan Medical School Institutional Review Board.

To support initial implementation, a member of the study team identified eligible patients each week by reviewing the log of upcoming appointments to assess age and diagnostic eligibility. A notation of “transition survey” was added to the EHR information about the scheduled appointment, to alert clinic staff that this was an EpiTRAQ‐eligible patient. The EHR also flagged eligibility for repeat EpiTRAQ completion at least 6 months after the prior date of completion.

Clinic check‐in staff provided a paper copy of the EpiTRAQ to identified patients and asked them to complete the form. When parents indicated their child was not cognitively capable of completing the form, parents were encouraged to complete the form, checking a box on the form to indicate parent‐reported data. EpiTRAQ forms were completed in the waiting area prior to the appointment or while waiting in the exam room. EpiTRAQ forms were collected at clinic check‐out.

### 2.4 Approach to validity and reliability assessment

In conjunction with the clinical implementation of EpiTRAQ, we evaluated the reliability and validity of the modified tool. Drawing on published guidance,^12^ we did not conduct a comprehensive factor analysis and validation of the revised instrument analysis of all items), including core TRAQ questions. First, we determined a priori that we would maintain all items in the established, validated TRAQ instrument and use a similar process for completion and scoring. Second, the additional epilepsy‐specific questions are clinically relevant and meaningful for this population as they were based on existing AAN epilepsy quality measures.

Therefore, our overall approach to EpiTRAQ validity and reliability assessment was to assess the internal consistency of the overall EpiTRAQ instrument and each subscale using Cronbach's alpha. We used the established TRAQ scoring protocol to generate the mean score and standard deviation for each subscale and for the overall instrument. We also compared EpiTRAQ to TRAQ, comparing mean scores for questions common to both instruments.

#### 2.4.1 Initial validation

Our initial validation used EpiTRAQ data collected from 302 patients seen in pediatric and adult epilepsy clinics between October 2017 and May 2018. Consistent with the original TRAQ validation, we included only patient‐completed forms. We used the established TRAQ scoring protocol to generate the mean score and standard deviation for each subscale and for the overall instrument. For the subscale *Managing Daily Activities,* we calculated the mean for the subset of nine questions targeted to all patients using data for the total population and recalculated the mean for the full subscale (including the two questions targeted to females) using data for female patients only. For the overall scale, we calculated the mean score for the subset of 33 questions targeted to all patients using data for the total population and recalculated the mean for all 35 questions (including the two questions targeted to females) using data for female patients only.

We assessed the internal consistency of each EpiTRAQ subscale and the overall tool using Cronbach's alpha.^13^ For the overall scale and the subscale *Managing Daily Activities,* we calculated the Cronbach's alpha for the subset of questions targeted to all patients using data for the total population and recalculated the Cronbach's alpha for the full subscale (including the 2 questions targeted to females) using data for female patients only.

We also conducted a parallel analysis of the 20 core TRAQ questions, comparing mean scores and internal consistency (Cronbach's alpha) for the overall scale and each subscale to the original published TRAQ validation.^8^

#### 2.4.2 Repeat validation

We conducted a second validation test for 381 patients who completed at least one EpiTRAQ between June 2018 and February 2019. For patients who completed more than one EpiTRAQ during that timeframe, we included data from the more recent form in the repeat validation. We calculated mean scores and assessment of internal consistency for the overall scale and each subscale, using parallel methods to the initial validation test as described above. We repeated the comparisons for the 20 core TRAQ questions to the original published TRAQ validation.

#### 2.4.3 Retest reliability

To assess the reliability of EpiTRAQ over time, we analyzed results patterns for 153 patients who completed two or more EpiTRAQ forms between October 2017 and May 2019. For patients with more than two forms, we compared the initial and the most recent form, determining for each question if the self‐reported score at Time 2 was lower, higher or the same as the score at Time 1. We calculated the total number of lower and higher scores for each patient and performed bivariate analyses using the chi‐square test to explore the association between the total number of lower and higher scores and the average time between patient age and the initial and repeat EpiTRAQ completion.

## 3 RESULTS

Table 2 presents the characteristics of the three validation populations.

**TABLE 2:** Characteristics of the validation populations

	Initial validation N = 302	Repeat validation N = 381	Retest reliability^a^ N = 153
Gender			
Female	54.6%	52.2%	54.9%
Male	45.4%	47.8%	45.1%
Age			
16‐18 y	31.1%	30.7%	28.8%
19‐21 y	27.5%	27.0%	29.4%
22‐26 y	41.4%	42.3%	41.8%
Clinic type			
Pediatric Neurology	30.1%	32.5%	29.4%
Adult Neurology	69.9%	67.5%	70.6%

^a^ Age and clinic type categorized based on earliest EpiTRAQ form.

### 3.1 Validation of EpiTRAQ

Table 3 presents the mean scale and subscale EpiTRAQ scores for the initial and repeat validation populations. Mean scores are slightly higher for repeat validation than initial validation but otherwise very comparable. The absolute value of differences comparing initial and repeat validations range from 0.05 to 0.1.

**TABLE 3:** Scoring and internal consistency in validation populations

Subscale (# of questions)	Initial validation		Repeat validation	
	Mean score (SD)	Cronbach's alpha	Mean score (SD)	Cronbach's alpha
Managing medications (6)	3.88 (0.97)	0.78	3.97 (0.92)	0.77
Appointment keeping (7)	3.58 (1.31)	0.92	3.68 (1.27)	0.92
Tracking health issues (9)	3.50 (1.01)	0.82	3.56 (1.00)	0.82
Talking with providers (2)	4.63 (0.75)	0.80	4.68 (0.72)	0.81
Managing daily activities				
All (9)	3.82 (0.89)	0.85	3.88 (0.91)	0.85
Females (11)	3.73 (0.87)	0.87	3.79 (0.90)	0.88
Overall				
All (33)	3.74 (0.87)	0.95	3.81 (0.84)	0.94
Females (35)	3.79 (0.80)	0.94	3.85 (0.81)	0.94

Table 3 also presents the assessment of EpiTRAQ’s internal consistency for the initial and repeat validation populations. The range of Cronbach's alpha values for both initial and repeat validation is (0.78, 0.95) and (0.77, 0.94), respectively. These capture ranges of good to high reliability within subscales and for the overall score.

### 3.2 Comparison of EpiTRAQ to TRAQ

Table 4 presents the comparison of mean scale and subscale scores for only the 20 questions included in the core TRAQ. Mean scores for initial and repeat validation show very small differences when compared with the mean score for the original TRAQ. The absolute value of differences in mean scores (TRAQ – EpiTRAQ) between the corresponding subscales and overall score of the original TRAQ and initial and repeat validation of the EpiTRAQ range from 0.01 to 0.22.

**TABLE 4:** Comparison of mean scores for core TRAQ questions

Subscale (# of questions)	Initial validation Mean (SD)	Repeat validation Mean (SD)	Original TRAQ^8^ Mean (SD)
Managing medications (4)	4.04 (1.05)	4.15 (0.99)	3.93 (1.07)
Appointment keeping (7)	3.58 (1.31)	3.68 (1.27)	3.57 (1.10)
Tracking health issues (4)	3.46 (1.17)	3.52 (1.15)	3.53 (1.10)
Talking with providers (2)	4.63 (0.75)	4.68 (0.72)	4.54 (0.93)
Managing daily activities (3)	4.31 (0.91)	4.35 (0.92)	4.33 (0.96)
Overall (20)	3.86 (0.95)	3.94 (0.91)	3.85 (0.98)

Table 5 presents the internal consistency for the 20 questions included in the core TRAQ, comparing the initial and repeat validation populations to the original TRAQ validation.^8^ The internal consistency is nearly identical across the three groups for the overall scale, and for subscales on appointment keeping and talking with providers. Internal consistency is similar across groups for the other subscales (differences of no more than 0.11).

**TABLE 5:** Comparison of internal consistency (Cronbach's alpha) for Core TRAQ Questions

Subscale (# of questions)	Initial validation Cronbach's alpha	Repeat validation Cronbach's alpha	Original TRAQ^7^ Cronbach's alpha
Managing medications (4)	0.76	0.75	0.86
Appointment keeping (7)	0.92	0.92	0.90
Tracking health issues (4)	0.73	0.72	0.77
Talking with providers (2)	0.80	0.81	0.80
Managing daily activities (3)	0.75	0.78	0.67
Overall (20)	0.94	0.93	0.94

### 3.3 Retest reliability

For the 153 unique patients included in the retest reliability analysis, the time between completion of the initial and repeat form ranged from 5 to 17 months, with 10.5% at 1‐5 months, 41.8% at 6‐11 months, and 47.7% at 12‐17 months.

Table 6 presents the proportion of patients who gave themselves a lower, higher, or same score at Time 2 compared to Time 1, for each EpiTRAQ. Overall, 30.1% had 0‐1 questions with a lower score at Time 2; 35.3% had 2‐4 questions with a lower score at Time 2; 24.2% had 5‐9 questions with a lower score at Time 2; and 10.5% had ≥10 questions with a lower score at Time 2. There were no significant associations between the number of questions with a lower Time 2 score and either age or time between completion of the initial and repeat form.

**TABLE 6:** Comparison of Time 2 vs Time 1 EpiTRAQ responses

	Lower (%)	Same (%)	Higher (%)
Can you name your seizure medications?	8.8	77.7	13.5
Do you take medications correctly and on your own?	6.8	81.8	11.5
Do you know what to do if you are having a bad reaction to your medications?	19.5	51.7	28.9
Do you fill a prescription if you need to?	11.3	61.3	27.3
Do you reorder medications before they run out?	14.1	54.9	31.0
Do you know how to use rescue medications to stop a long or back‐to‐back seizure?	15.9	52.2	31.9
Do you call the doctor's office to make an appointment?	17.7	53.1	29.3
Do you follow‐up on any referral for tests, check‐ups or labs?	14.7	52.0	33.3
Do you arrange for your ride to medical appointments?	9.5	70.8	19.7
Do you call the doctor about unusual changes in your health (For example: An increase in seizure activity or allergic reactions)?	14.3	55.8	29.9
Do you apply for health insurance if you lose your current coverage?	17.2	48.5	34.3
Do you know what your health insurance covers?	18.9	44.1	37.1
Do you manage your money & budget household expenses (For example: use checking/debit card)?	15.7	49.0	35.4
Can you explain what type of seizures you have?	16.2	62.8	21.0
Can you explain why you have epilepsy?	18.3	51.1	30.7
Do you know what to do if you know that you are going to have a seizure?	15.3	54.9	29.9
Do you know how to keep track of your seizures?	11.7	60.0	28.3
Do you know how a seizure action plan is used?	16.3	45.4	38.3
Do you fill out the medical history form, including a list of your allergies?	10.8	62.2	27.0
Do you keep a calendar or list of medical and other appointments?	16.3	52.4	31.3
Do you make a list of questions before the doctor's visit?	25.0	33.1	41.9
Do you get financial help with school or work?	23.3	50.0	26.7
Do you tell the doctor or nurse what you are feeling?	8.3	73.8	17.9
Do you answer questions that are asked by the doctor, nurse, or clinic staff?	4.8	83.5	11.7
Do you help plan or prepare meals/food?	13.9	62.8	23.4
Do you keep home/room clean or clean‐up after meals?	14.3	62.9	22.9
Do you use neighborhood stores and services? (For example: Grocery stores and pharmacy stores?)	9.9	73.9	16.2
Can you explain how epilepsy affects school or having a job?	8.6	69.3	22.1
Can you explain activities that you are not allowed to do?	14.2	68.1	17.7
Can you describe how long you have to be seizure free before you are allowed to drive a car?	8.5	79.6	12.0
Can you describe how other medications and alcohol affect your seizure medications?	17.3	58.3	24.5
Can you describe how seizures can affect your ability to have children?	14.5	46.4	39.1
Can you describe how epilepsy and seizure medications affect bone health?	13.5	46.1	40.4
If you become pregnant, can you explain how seizures affect your pregnancy? *(Female only)*	12.8	47.4	39.7
Can you explain how seizure medications and birth control medications affect one another? *(Female only)*	20.5	42.3	37.2

The majority of patients rated themselves with higher scores: 17% had 0‐2 questions with a higher score at Time 2; 40.5% had 3‐9 questions with a higher score at Time 2; and 41.8% had ≥10 questions with a higher score at Time 2. Younger age was associated with reporting higher scores at Time 2:56.8% of patients 16‐18 years reported ≥10 questions with a higher score at Time 2, compared to 44.4% of patients 19‐21 and 29.7% of patients ≥22 years (*P* = .005). There was no significant association between the number of questions with a higher Time 2 score and time between completion of the initial and repeat form.

## 4 DISCUSSION

Transition from pediatric to adult care should be based on readiness, not simply age‐eligibility criteria. For patients with epilepsy, lack of or poor transition may be associated with suboptimal seizure control, increased risk of sudden unexpected death in epilepsy (SUDEP), increased social isolation.^4^, ^14^ Thus, an epilepsy‐specific transition readiness assessment tool is important to support the unique transition needs of this population, including condition‐specific knowledge and information that are not covered in general assessments.^4^, ^5^

Many transition readiness assessment tools are geared toward the primary care setting. However, primary care pediatricians may feel unprepared to discuss disease‐specific issues related to young adulthood, such as reproductive health issues.^15^ In contrast, physicians and other clinical staff in the specialty neurology clinic are well prepared to discuss epilepsy‐specific information and management strategies for youth preparing for the adult system of care. An epilepsy‐specific transition readiness assessment tool appropriate for implementation in the neurology clinic setting may serve to facilitate such discussions.

In working toward an epilepsy‐specific tool, we chose to make incremental changes to a widely used instrument to improve its relevance for a target population. Thus, EpiTRAQ is grounded within the well‐validated TRAQ instrument. TRAQ has been used in other condition‐specific populations and has been translated into multiple languages.^6^ TRAQ’s broad use is evidence of its value to providers, patients, and parents. Another strength of EpiTRAQ is its close relationship to AAN quality measures of recommended healthcare delivery and important outcomes for youth with epilepsy.^10^, ^11^ Quality measures are developed from evidence‐based published information where a gap in implementation of this evidence exists. The close correspondence of EpiTRAQ to the AAN quality measures reinforces the validity of the tool and may represent a rationale for adoption in neurology clinics.

We prioritized ease of clinical implementation in developing and testing EpiTRAQ. Although the process of identifying patients eligible for EpiTRAQ was done manually at the outset of implementation, we quickly moved toward use of the EHR for this purpose. Similarly, initial completion of EpiTRAQ was done on paper copies; we plan to add the option for EpiTRAQ completion in electronic form via our patient portal, with direct entry into the patient's EHR. Finally, our experience may represent an option for other clinics that want to incorporate specialty‐specific items into a general transition readiness assessment tool.

Results of our EpiTRAQ validation demonstrated that mean scores compared favorably to the original TRAQ validation^8^, for both the initial and repeat validation populations. Among the three subscales with new epilepsy‐specific questions, mean scores for Managing Medications were higher, Tracking Health Issues were slightly lower, and Managing Daily Activities equivalent to the mean scores from the original TRAQ validation. Mean scores for the two subscales without new questions (Appointment Keeping, Talking with Providers) were higher than those in the original TRAQ validation. Internal consistency for EpiTRAQ was also comparable to the original TRAQ, with all but one subscale achieving a Cronbach's alpha of high (≥0.90) or very good (0.8‐0.89), with the remaining subscale as good (0.70‐0.79).^13^ Moreover, internal consistency for two of the three subscales with new epilepsy‐specific questions (Tracking Health Issues, Managing Daily Activities) was higher for EpiTRAQ than for the original TRAQ validation; in contrast, internal consistency for Managing Medications was lower for EpiTRAQ than the original TRAQ validation. Internal consistency for the two subscales without new questions (Appointment Keeping, Talking with Providers) was nearly identical to the original TRAQ validation.^8^

Finally, retest reliability of EpiTRAQ was strong. Over 80% of adolescent and young adult patients gave themselves higher ratings on at least 3 items at Time 2, which occurred at least 6 months later than Time 1, and younger age was associated with an increase in scores from Time 1 to Time 2s. In this population of patients 16‐26 years of age, we might expect more opportunities to increase knowledge and confidence in younger patients, who may be newly encountering the self‐management and knowledge concepts reflected in the EpiTRAQ items. This would be consistent with the original TRAQ validation, which showed higher scores among older patients.^8^ Still, over one third of adolescent and young adult patients gave themselves lower ratings on at least 5 items at Time 2. It is unknown whether this reflects a true decrease in patients’ knowledge or self‐management skills, or perhaps an emerging awareness that they are not confident about certain topics.

There are several limitations of EpiTRAQ. First, the established response structure for TRAQ does not include an option for “not applicable”; this may have been useful for patients who are not currently using medications related to their epilepsy. Second, consistent with the original TRAQ, we did not include parent‐reported responses in this validation exercise. However, a considerable proportion of youth with epilepsy have cognitive deficits; as such, further work to understand the value of EpiTRAQ for parents of this subset of epilepsy patients is warranted. Third, we targeted EpiTRAQ to ages 16‐26; while this is consistent with TRAQ, it should be noted that EpiTRAQ was not designed to assess transition readiness among younger adolescents who are just beginning the transition process. Finally, there is a lack of research demonstrating a link between transition readiness assessment results and clinical outcomes, both for EpiTRAQ and the original TRAQ; this may be, in part, due to the lack of metrics that determine transition success.^4^ There is a need for additional research to understand the link between EpiTRAQ and patient outcomes.

Overall, EpiTRAQ may be a valid and reliable tool for assessing transition readiness in adolescents and young adults with epilepsy without major cognitive deficit or intellectual disability. Additional work is needed to explore the association with longitudinal changes in EpiTRAQ scores and clinical outcomes over time.

## CONFLICTS OF INTEREST

The authors received salary support through the abovementioned HRSA grant and have no other conflicts of interest to disclose. The authors confirm we have read the Journal's position on issues involved in ethical publication and affirm that this report is consistent with those guidelines.
